# MMP24 Contributes to Neuropathic Pain in an FTO-Dependent Manner in the Spinal Cord Neurons

**DOI:** 10.3389/fphar.2021.673831

**Published:** 2021-04-29

**Authors:** Longfei Ma, Yangyuxin Huang, Fengjiang Zhang, Dave Schwinn Gao, Na Sun, Jinxuan Ren, Suyun Xia, Jia Li, Xinyi Peng, Lina Yu, Bao-Chun Jiang, Min Yan

**Affiliations:** ^1^ Department of Anesthesiology, Second Affiliated Hospital of Zhejiang University School of Medicine, Hangzhou, China; ^2^ Institute of Pain Medicine and Special Environmental Medicine, Nantong University, Nantong, China

**Keywords:** neuropathic pain, matrix metallopeptidase 24, fat-mass and obesity-associated protein, N6-methyladenosine, spinal cord

## Abstract

Nerve injury-induced gene expression change in the spinal cord is critical for neuropathic pain genesis. RNA N^6^-methyladenosine (m^6^A) modification represents an additional layer of gene regulation. We showed that spinal nerve ligation (SNL) upregulated the expression of matrix metallopeptidase 24 (MMP24) protein, but not *Mmp24* mRNA, in the spinal cord neurons. Blocking the SNL-induced upregulation of spinal MMP24 attenuated local neuron sensitization, neuropathic pain development and maintenance. Conversely, mimicking MMP24 increase promoted the spinal ERK activation and produced evoked nociceptive hypersensitivity. Methylated RNA Immunoprecipitation Sequencing (MeRIP-seq) and RNA Immunoprecipitation (RIP) assay indicated the decreased m^6^A enrichment in the *Mmp24* mRNA under neuropathic pain condition. Moreover, fat-mass and obesity-associated protein (FTO) was colocalized with MMP24 in spinal neurons and shown increased binding to the *Mmp24* mRNA in the spinal cord after SNL. Overexpression or suppression of FTO correlates with promotion or inhibition of MMP24 expression in cultured spinal cord neurons. In conclusion, SNL promoted the m^6^A eraser FTO binding to the *Mmp24* mRNA, which subsequently facilitated the translation of MMP24 in the spinal cord, and ultimately contributed to neuropathic pain genesis.

## Introduction

Nerve injury-induced neuropathic pain is a refractory and debilitating disease (Mitka, 2003). Limited treatments are available for this disorder because most therapies work only symptomatically via neurotransmission blockade but ignore the underlying pain pathogenesis and its phasic progression. The development of more efficient treatment of this disease comes to a standstill due to our incomplete understanding of mechanisms underlying the neuropathic pain induction and maintenance (Ji et al., 2009). Abnormal gene expression changes in the dorsal root ganglion (DRG) and spinal cord are the crucial molecular basis for developing and maintaining neuropathic pain (Jiang et al., 2016; Pokhilko et al., 2020).

Epigenetic processes, such as DNA methylation, histone modifications, and non-coding RNAs, frequently regulate pain-related gene expression (Jiang et al., 2018; Wu et al., 2019; Lin et al., 2020). RNA methylation mediated RNA post-transcriptional modification has recently been emphasized as a significant epigenetic modification (He and He, 2021). N6-methyladenosine (m^6^A) is the most prevalent and dynamic modification in mRNA with a preference for the 3′-untranslated region (3′-UTR) and the transcription starting site (Dominissini et al., 2012; Meyer et al., 2012; Fu et al., 2014). m^6^A is installed by “writer” complex composed of methyltransferase-like 14 (METTL14), methyltransferase-like 3 (METTL3) and Wilms’ tumor 1-associating protein (WTAP), and removed by “eraser” demethylases fat-mass and obesity-associated protein (FTO) and AlkB homolog 5 (ALKBH5) (Dominissini et al., 2012; Zheng et al., 2013; Fu et al., 2014; Liu et al., 2015). This modification is recognized by m^6^A-binding proteins (Dominissini et al., 2012; Fu et al., 2014; Liu et al., 2015) to mediate various mRNA biogenesis, such as RNA stability, translation, splicing and export (Yang et al., 2018). In particular, recent evidence revealed that neuropathic pain could be partially attributed to the methyltransferases/demethylases-induced RNA m^6^A modification dysregulation of pain-associated genes in the DRG (Li et al., 2020; Albik and Tao, 2021). However, the role of spinal m^6^A modification in the neuropathic pain genesis remains largely unknown.

Increasing evidence suggests that proteases such as matrix metalloproteases (MMPs), caspases, and cathepsin S play a vital role in the genesis of neuropathic pain by regulating neuroinflammation in the central nervous system (Ji et al., 2009; Hannocks et al., 2019; Jiang et al., 2020a), through the cleavage of the extracellular matrix proteins, chemokines, and cytokines (Manicone and McGuire, 2008). The MMP family includes more than 20 members. Among them, MMP2 and MMP9 are involved in the regulation of neuropathic pain during the early and late phase, respectively, through the cleavage/activation of interleukin 1 beta (IL-1β) in the extracellular matrix and subsequent phosphorylation of extracellular signal-regulated kinase 1/2 [pERK1/2, a marker for spinal neuron hyper-sensitization (Zhuang et al., 2005)] (Kawasaki et al., 2008). MMP24 is abundantly expressed throughout the nervous system (Hayashita-Kinoh et al., 2001) and could degrade several extracellular matrix components, including cell-adhesion molecule N-cadherin to promote neurite outgrowth in cultured cells (Hayashita-Kinoh et al., 2001). Mouse strain lacking in MMP24 was found absent of thermal hyperalgesia with inflammation model (Folgueras et al., 2009). However, whether MMP24 in the spinal cord participates in the development or maintenance of neuropathic pain is still elusive.

Here, we report that spinal nerve ligation (SNL) leads to a significant increase in the level of MMP24 protein, but not *Mmp24* mRNA, in the spinal cord. This increase contributes to the SNL-induced neuropathic pain induction and maintenance, possibly by the activation of ERK1/2. Mechanistically, SNL promoted FTO binding to the *Mmp24* mRNA for the erasure of m^6^A enrichment and possibly accelerated the *Mmp24* mRNA translation in the spinal cord.

## Materials and Methods

### Animals

Male C57BL/6J mice (6–8 weeks old for *in vivo* experiments) were purchased from SLAC Laboratory (Shanghai, CN). Rodents were hosted in a centralized location in the Second Affiliated Hospital of Zhejiang University (SAHZU), School of Medicine, with a standardized circadian cycle with 12 h of light and darkness. Mouse chow and water were provided, ad libitum. All experiments were approved by the Zhejiang Animal Care and Use Committee and the Ethics Committee of SAHZU, School of Medicine. Utmost care was taken to ensure the welfare of the rodents and kept their usage to a minimum. Behavioral experiments were undertaken with blinded investigators, with no knowledge of the viral content or other preparatory conditions.

### Animal Model

L4 spinal nerve ligation (SNL) was carried out according to the methods previously described (Decosterd and Woolf, 2000; Wang and Wang, 2003; Rigaud et al., 2008). In brief, mice were anesthetized with Nembutal. The lower back was dissected until the transverse lumbar process was exposed. After the process was removed, the underneath L4 spinal nerve was ligated with a silk 6–0 thread. A slight distal location was chosen for transection around the ligation site. Subsequent layers of muscle and skin were closed. The sham groups undertook identical procedures, but without the transection or ligature of the corresponding nerve.

### Behavioral Tests

Mechanical and thermal pain tests along with locomotor function assessments were performed as described in the previous studies (He et al., 2011; Zhao et al., 2013; Daskalaki et al., 2018). Each behavioral test was carried out at 1 h interval.

Paw withdrawal frequencies (PWF) were defined as a response to physical stimulation via von Frey filaments. In short, the animal was introduced to an individual Plexiglas chamber on an elevated mesh screen. Two calibrated von Frey filaments (0.07 and 0.4 g) (DanMic Global. Campbell, CA) were utilized to stimulate the hind paw for ∼1 s and the hind paw was stimulated repeatedly 10 times at 5 min interval. Paw withdrawal responses were aggregated, averaged over the number of trials, and calculated in percentage, which resulted in the PWF ((number of paw withdrawals/10 trials) × 100 = % response frequency).

A Model 336 Analgesia Meter (IITC Inc. Life Science Instruments. Woodland Hills, CA) was utilized to measure paw withdrawal latencies (PWL) to the noxious application of heat. In short, the rodent was introduced to an individual Plexiglas chamber on a glass plate. The analgesic meter's light emitter beamed through a keyhole onto the hind paw’s plantar surface, with the stimuli switched off upon paw withdrawal. The paw withdrawal latency was logged, defined as time passed between initiation of the stimuli to paw withdrawal. Five trials were undertaken with an interval of 5 min each. 20 s cut-off limit was defined to eliminate any tissue injury.

Locomotor function tests the righting, grasping, and placing reflexes after PWF and PWL. Righting reflex: rodent placed supine on a flat surface, the time it takes to upright itself to a normal position was recorded. Grasping reflex: rodent placed on a meshed wire, any contact or grasp of the wire was recorded. Placing reflex: rodent placed on the edge of a surface with the hind leg in a lower position than forelimbs, while leaving hindlimbs just off contact with the edge of a platform, and recorded whether hind paws reflexively placed on the platform. Each trial was repeated at a 5 min interval five times and the scores for each reflex were recorded on the basis of counts of each normal reflex.

### Intraspinal and Intrathecal Injection

The intraspinal injection was performed as described previously (Jiang et al., 2016). In short, after anesthetized with Nembutal, mice underwent hemilaminectomy at the L1-L2 vertebral segments. The intraspinal injection was carried out ipsilaterally on the left side. By using a glass micropipette, each animal received two injections (5 × 10^5^ TU per injection, 0.8 mm from the midline, 0.5 mm apart in rostrocaudal axis, 0.5 mm deep) of lentivirus following the L3-L4 dorsal root entry zone after exposure of spinal cord. The tip of glass micropipette should reach a depth of lamina II-IV of the spinal cord. Finally, the dorsal muscle and skin were sutured layer by layer.

Intrathecal injection of siRNA (20 μM, 10 μL) was performed daily for two to three consecutive days in sham or SNL mice. Mice were held firmly in place over the pelvic girdle and between L5 and L6 vertebrae inserted a 30-gauge needle attached to a 25 μL microsyringe. A slight flick of the tail after a sudden advancement of the needle confirmed the proper insertion of the needle into the subarachnoid space (Jiang et al., 2016). TurboFect *in vivo* transfection reagent (Thermo Fisher, R0541) was used to improve delivery and prevent siRNA degradation. *Mmp24* siRNA1 (sense: 5′-GAG AUU CGU CUU CUU CAA ATT-3′, antisense: 5′-UUU GAA GAA GAC GAA UCU CTT-3′), *Mmp24* siRNA2 (sense: 5′-GGA UAU UAC ACC UAC UUC UTT-3′, antisense: 5′-AGA AGU AGG UGU AAU AUC CTT-3′), *Mmp24* siRNA3 (sense: 5′-CUA UCU UCC AAU UCA AGA ATT-3′, antisense: 5′-UUC UUG AAU UGG AAG AUA GTT-3′).

### Spinal Dorsal Horn Neuron Culture and Viral Transduction

Primary spinal neuronal cultures were prepared from 1 to 2 week old C57BL/6J mice using a procedure modified from our previously described method (Jiang et al., 2016). In short, a laminectomy was conducted and the spinal cord was carefully removed after decapitation. Superficial dorsal horn was isolated and then cut into several strips. The strips were incubated at 37°C for 45 min in Hanks’ balanced salt solution (HBSS, Invitrogen) containing papain (15 U/ml, Worthington Biochemical), then rinsed 3 times with HBSS, and placed in mixed Neurobasal Medium (Invitrogen) containing 5% fetal bovine serum (FBS, Invitrogen), heat-inactivated horse serum (5%, Invitrogen), B-27 (1%, Invitrogen), L-glutamax-1 (2 mM, Invitrogen), streptomycin (100 μg/ml, Invitrogen) and penicillin (100 U/ml, Invitrogen). A Fire-polished Pasteur pipette was used to dissociate fragments by gentle trituration mechanically. The resulting cell suspension was plated onto 6-well plates coated with poly-d-lysine and collagen. The cells were incubated at 37°C with 95% O_2_, 5% CO_2_. After the neurons were treated with cytosine arabinoside, 2–10 μL virus (titer ≥1 × 10^13^) was added to each well. The neurons were collected 3 days after virus transduction.

### Reverse Transcription (RT)-PCR

Total RNA from the cultured samples or tissue was extracted and purified using miRNeasy kit with genome DNA Eliminator Columns (QIAGEN, Germany). The SuperScript^™^ First-Strand Synthesis System (Invitrogen/Thermo Fisher) was then used to reverse-transcribe RNA. Each sample was run in a 20 μL reaction with 20 ng cDNA, 10 µL SsoAdvanced Universal SYBR Green Supermix (Bio-Rad Laboratories, CA) and 250 nM forward and reverse primers. *Tuba1a* was used as an internal control for normalization. Real-time PCR was performed on the Applied Biosystems QuantStudio5 system (Applied Biosystems, CA). ΔCt method (2^−ΔΔCt^) was applied for mRNA levels calculation. All data were normalized to *Tuba1a*, which has been demonstrated to be stable even after peripheral nerve injury insult (Zhao et al., 2013; Wu et al., 2016). All the primers are listed in Table 1.

**TABLE 1:** Primers sequence for qRT-PCR or RIP-PCR.

Gene	Sequences	Size
*Mmp24*	F 5′-TGA​CCC​CAG​TGC​TAT​CAT​GG-3′	176 bp
	R 5′-TCT​CAG​ATG​GCG​AGT​GGA​TC-3′	
*Fto*	F 5′-GTG​AGG​ACG​AGT​CCA​GCT​TC-3′	225 bp
	R 5′-AGG​TGC​CTG​TTG​AGC​ACT​CT-3′	
*Mettl3*	F 5′-AAG​GAG​CCG​GCT​AAG​AAG​TC-3′	248 bp
	R 5′-TCA​CTG​GCT​TTC​ATG​CAC​TC-3′	
*Mettl14*	F 5′- TGA​GAG​TGC​GGA​TAG​CAT​TG-3′	200 bp
	R 5′-TCT​CTT​CCT​CCT​GCT​GCA​TT-3′	
*Alkbh5*	F 5′-GGA​ACC​TGT​GCT​TTC​TCT​GC-3′	176 bp
	R 5′-TGC​TCA​GGG​ATT​TTG​TTT​CC-3′	
*Wtap*	F 5′-GCA​AGA​GTG​CAC​CAC​TCA​AA-3′	161 bp
	R 5′-CAT​TTT​GGG​CTT​GTT​CCA​GT-3′	
*Tuba1a*	F 5′-GTG​CAT​CTC​CAT​CCA​TGT​TG-3′	210 bp
	R 5′-GTG​GGT​TCC​AGG​TCT​ACG​AA-3′	

### Plasmids Construction and Virus Production

The *Mmp24* and *Fto* coding sequences were synthesized by Tsingke Biological Technology (Beijing, CN), and were further inserted into pro-viral plasmids pLV-CMV-MCS-Ubi-ZSGreen and pAAV-CMV-MCS-F2A-EGFP, respectively, using the Seamless Cloning and Assembly Kit (SunBio, CN). pLV-CMV-ZSGreen or pAAV-CMV-EGFP was used as the corresponding control (SunBio, CN). Lentiviruses and adeno-associated viruses packaging were completed by the Tsingke Biological Technology (Beijing, CN) and Sunbio Technology (Shanghai, CN). The *Fto* coding region's shRNA (GenBank accession number NM_011936.2) was designed to target the sequence 5′-GTC TCG TTG AAA TCC TTT GAT-3′. A scramble shRNA was used as control (5′-TTC TCC GAA CGT GTC ACG T-3′). Both *Fto* and scrambled shRNA oligonucleotides were inserted to pAAV-CAG-EGFP-U6-shRNA and packaged into adeno-associated virus by SunBio. All the constructs were sequenced to prove sequence integrity.

### Immunohistochemistry

After anesthetized with Nembutal, mice were perfused with cold PBS and 4% paraformaldehyde through the ascending aorta. Following perfusion, the L4 spinal cord segments were removed, post-fixed in the same fixative at 4°C overnight and dehydrated. Spinal cord sections (25 μm) were cut in a cryostat and processed for immunofluorescence. The sections were blocked for 1 h at room temperature in PBS containing 5% goat or donkey serum and then incubated overnight at 4°C with the following primary antibodies: mouse anti-FTO (1:100, Abcam), rabbit anti-MMP24 (1:100, Affinity), mouse anti-NeuN (1:500, Abcam), rabbit anti-NeuN (1:500, Abcam), mouse anti-GFAP (1:500, EMD Millipore), rabbit anti-GFAP (1:500, Abcam), mouse anti-IBA1 (1:200, Sigma), rabbit anti-IBA1 (1:200, Wako). After wash, the sections were then incubated with corresponding second antibodies with either Alexa FluorTM 488- or Alexa FluorTM 594-labeled (1:500, Invitrogen) at room temperature for 1 h. 4′, 6-diamidino-2-phenylindole (DAPI) (Abcam) was finally utilized for slides mounting. Leica DMI4000 fluorescence microscope was used for immunofluorescence-labeled image examination (Leica, Germany).

### Western Blotting

Cytosolic and nuclear proteins were extracted from the L4 spinal cord as previous study (Zhao et al., 2017). Ice-cold lysis buffer was utilized for the tissue homogenization, which contained 10 mM Tris, 5 mM EGTA, 2 mM MgCl_2_, 1 mM DTT, 1 mM phenylmethylsulfonyl fluoride, and 40 μM leupeptin. After centrifugation (4°C, 15 min, 1,500 g), the supernatants were gathered for cytosolic proteins and the pellets for nuclear proteins. The protein sample concentration was measured using Detergent Compatible Bradford Protein Assay Kit (Beyotime, CN). After heated at 99°C for 5 min, the samples were loaded onto an SDS-polyacrylamide gel (Genshare Biology, CN) and then electrophoretically transferred onto a polyvinylidene fluoride membrane (Millipore, Burlington, MA, United States). The membranes were blocked with 5% nonfat milk TBST for 1 h and then incubated overnight with the following antibodies: mouse anti-FTO antibody (1:1,000, Abcam), rabbit anti-METTL3 antibody (1:1,000, Abcam), rabbit anti-METTL14 antibody (1:1,000, Proteintech), rabbit anti-ALKBH5 antibody (1:1,000, Abcam), mouse anti-WTAP antibody (1:100, Santa Cruz), rabbit anti-MMP24 antibody (1:1,000, Affinity), mouse anti-histone H3 (1:3,000, Santa Cruz), mouse anti-GAPDH (1:1,000, Zhongshan Golden Bridge Bio-technology), rabbit anti-phospho-ERK1/2 (Thr202/Thy204, 1:2,000, CST), rabbit anti-ERK1/2 (1:2,000, CST). Horseradish peroxidase-conjugated anti-mouse or rabbit secondary antibody (1:5,000, Jackson ImmunoResearch) were utilized for protein detection and Clarity Western ECL Substrate (EMD Millipore) for visualization and ChemiDoc XRS + System (Bio-Rad) for exposure. NIH ImageJ software was utilized for quantification of the intensity of blots with densitometry. After each was normalized to the corresponding GAPDH or histone H3 (for nucleus proteins), the relative density values of the treated groups or different time points were determined by dividing the optical density values from these groups by the average value of the naïve/control groups.

### RNA Immunoprecipitation Assay

Magna RIP Kit (EMD Millipore, Darmstadt, Germany) was used for the RNA Immunoprecipitation (RIP) assay. The spinal cord homogenates were suspended in the RIP lysis buffer containing RNase inhibitor and protease inhibitor cocktail. The RIP lysate was incubated on ice for 5 min and kept in −80°C. Use the RIP wash buffer to wash Magnetic Beads Protein A/G suspension for each IP. Magnetic Beads Protein A/G re-suspended in RIP wash buffer were incubated with Mouse anti-FTO antibody (4 μg; Santa Cruz), mouse anti-m^6^A antibody (4 μg; Abcam), or normal mouse IgG for 30 min at room temperature. The Beads Protein A/G-antibody complexes were re-suspended into the RIP immunoprecipitation buffer after being washed three times with RIP wash buffer. The RIP lysate supernatants were incubated with beads-antibody complex in the RIP buffer overnight at 4°C by rotating after being thawed and centrifuged at 14,000 rpm for 10 min at 4°C. RIP wash buffer was used to wash the samples six times. After incubating the beads in the proteinase K buffer at 55°C for 30 min by shaking, the RNA was eluted and purified by phenol/chloroform extraction from the beads. The RNA enrichment was analyzed by quantitative real-time PCR. The RIP lysate supernatant was used as input. All primers are listed in Table 1.

### Methylated RNA Immunoprecipitation Sequencing (MeRIP-Seq) and MeRIP-Seq Libraries

Following the manufacturer’s procedure, Trizol reagent (Invitrogen, CA, United States) was used to extract total RNA. Bioanalyzer 2,100 and RNA 6,000 Nano LabChip Kit (Agilent, CA, United States) were utilized to analyze the total RNA quality and quantity with RIN number >7.0. To isolate Poly (A) mRNA, nearly more than 200 ug of total RNA were gathered and mixed with poly-T oligo attached magnetic beads (Invitrogen). After purification, divalent cations were applied to fragment the poly(A) mRNA fractions into 50–150 nt oligonucleotides under elevated temperature. The cleaved RNA fragments were then incubated in IP buffer (0.5% Igepal CA-630, 50 mM Tris-HCl and 750 mM NaCl) supplemented with BSA (0.5 μg/μL) for 2 h at 4°C with m^6^A-specific antibody (No.202003, Synaptic Systems, Germany). The mixture was then incubated together with protein-A beads and eluted with elution buffer (6.7 mM m^6^A and 1 × IP buffer). 75% ethanol was used to precipitate the eluted RNA. In conformity to a strand-specific library preparation by dUTP method, untreated input control fragments and eluted m^6^A-containing fragments (IP) are converted to the final cDNA library. The average insert size for the paired-end libraries was ∼100 bp. Following the vendor's recommended protocol, the paired-end 2 × 100 bp sequencing was then carried out on an Illumina Novaseq^™^ 6,000 platform at the LC-BIO Bio-tech Ltd. (Hangzhou, CN).

Firstly, to remove the reads that contained adaptor contamination, undetermined bases and low-quality bases, in-house Perl scripts and Cutadapt (Kechin et al., 2017) were applied. By using FastQC (http://www.bioinformatics.babraham.ac.uk/projects/fastqc/), the sequence quality was further validated. To map reads, bowtie (Langmead and Salzberg, 2012) were utilized to reference genome with default parameters. Mapped reads are then provided as input for MACS2 (Zhang et al., 2008), which helps to identify m^6^A peaks that can be adapted for visualization on the UCSC genome browser. *De novo* motif finding is carried out by using MEME (Bailey et al., 2009), followed by localization of the motif in respect of peak summit by in-house Perl scripts. By using ChIPseeker (Yu et al., 2015), called peaks are annotated by the intersection with gene architecture. MeRIP-seq data files were submitted to the GEO repository through the access code: GSE171004.

### Statistical Analysis

The cells were suspended evenly and dispersed randomly in each well for *in vitro* trials. The animals were randomly distributed into various treatment groups for *in vivo* experiments. All of the results were specified as mean ± SEM. For the biochemical results, two-tailed, unpaired Student's *t*-test (two groups) and one-way ANOVA (> 2 groups) were utilized for the data analyses. For the behavioral results, two-way ANOVA was used for the data analyses. Pairwise comparisons between means were tested by the *post hoc* Tukey method (GraphPad Prism 8) when ANOVA showed significant differences. The detailed analyzed process utilized in each experiment was elaborated in the matching figure legends. The sample sizes were determined based on the previous reports, pilot studies in the field and power analyses (power of 0.90 at *p* < 0.05) (Ferreira et al., 2001; Zhao et al., 2013; Millard et al., 2016; Daskalaki et al., 2018; Sato et al., 2018). Significance was set at *p* < 0.05.

## Results

### MMP24 Protein Upregulation in the Spinal Cord after SNL

**FIGURE 1:**
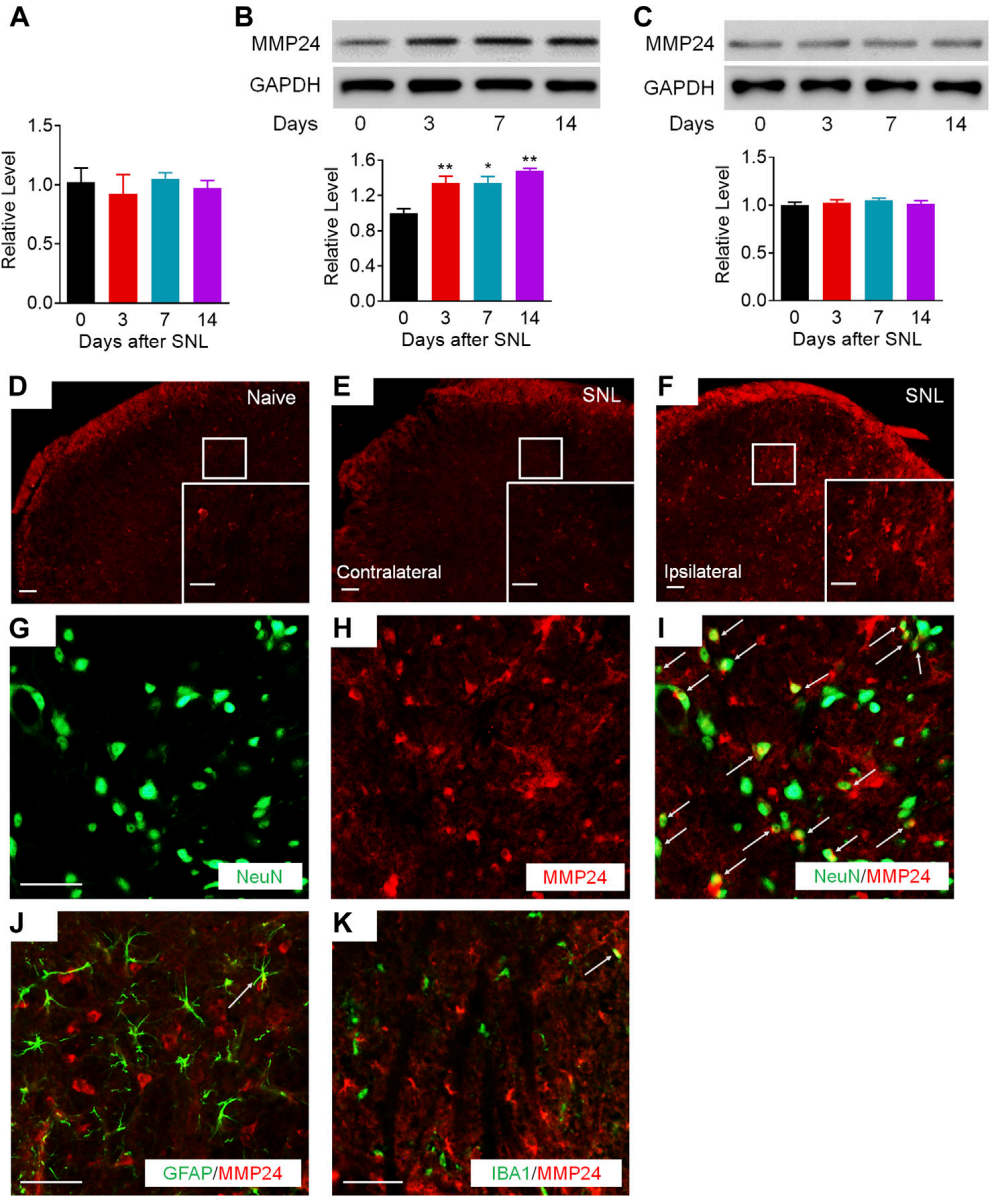
MMP24 protein is increased in the spinal cord after SNL **(A)**. Expression of *Mmp24* mRNA after SNL. *n* = 4 mice/group/time point. **(B)**. Expression of MMP24 protein in the spinal cord after SNL surgery. *n* = 3–6 mice/time point. One-way ANOVA followed by *post hoc* Tukey test. F_time_ (3, 8) = 29.00. ^*^ *p* < 0.05, ^**^ *p* < 0.01 vs. the corresponding control group (0 day). **(C)**. Expression of MMP24 protein in the spinal cord after sham surgery. *n* = 3 mice/time point. **(D–F)**. Representative images of MMP24 immunofluorescence in the L4 dorsal horn. MMP24 immunoreactivity was low in naïve mice, but increased in the ipsilateral dorsal horn compared with the contralateral dorsal horn 7 days after SNL. Scale bar: 50 μm; 25 μm (insets). **(G–K)**. Double staining of MMP24 and markers of neuron, astrocytes, and microglia on day 14 after SNL. Scale bar: 50 μm.

We first examined whether the MMP24 enrichment was altered in the spinal cord after SNL, a preclinical animal model that mimics nerve injury-induced neuropathic pain in the clinical setting (Rigaud et al., 2008). Unilateral SNL did not alter the level of *Mmp24* mRNA in the spinal cord from day 3 to 14 post-SNL (Figure 1A). However, the expression of MMP24 protein was significantly increased in a time-dependent manner in the spinal cord after SNL (Figure 1B), but not after sham surgery (Figure 1C). Immunostaining further confirmed that the MMP24 protein was dramatically increased in the ipsilateral dorsal horn, but not in the contralateral dorsal horn, on day 7 post-SNL (Figures 1D–F). The distribution of MMP24 in the spinal dorsal horn was also checked by double immunostaining with different cell markers. As shown in Figures 1G–K, MMP24 was predominantly colocalized with the neuronal-specific nuclear protein (NeuN) (Figures 1G–I), and rarely with the glial fibrillary acidic protein (GFAP) (Figure 1J), or ionized calcium-binding adaptor molecule (IBA-1) (Figure 1K) in the spinal dorsal horn on day 14 after SNL. The above evidence suggests that the increased MMP24 protein in the spinal dorsal horn may involve neuropathic pain.

### Blocking Spinal MMP24 Increase Attenuates Neuropathic Pain Development and Maintenance

**FIGURE 2:**
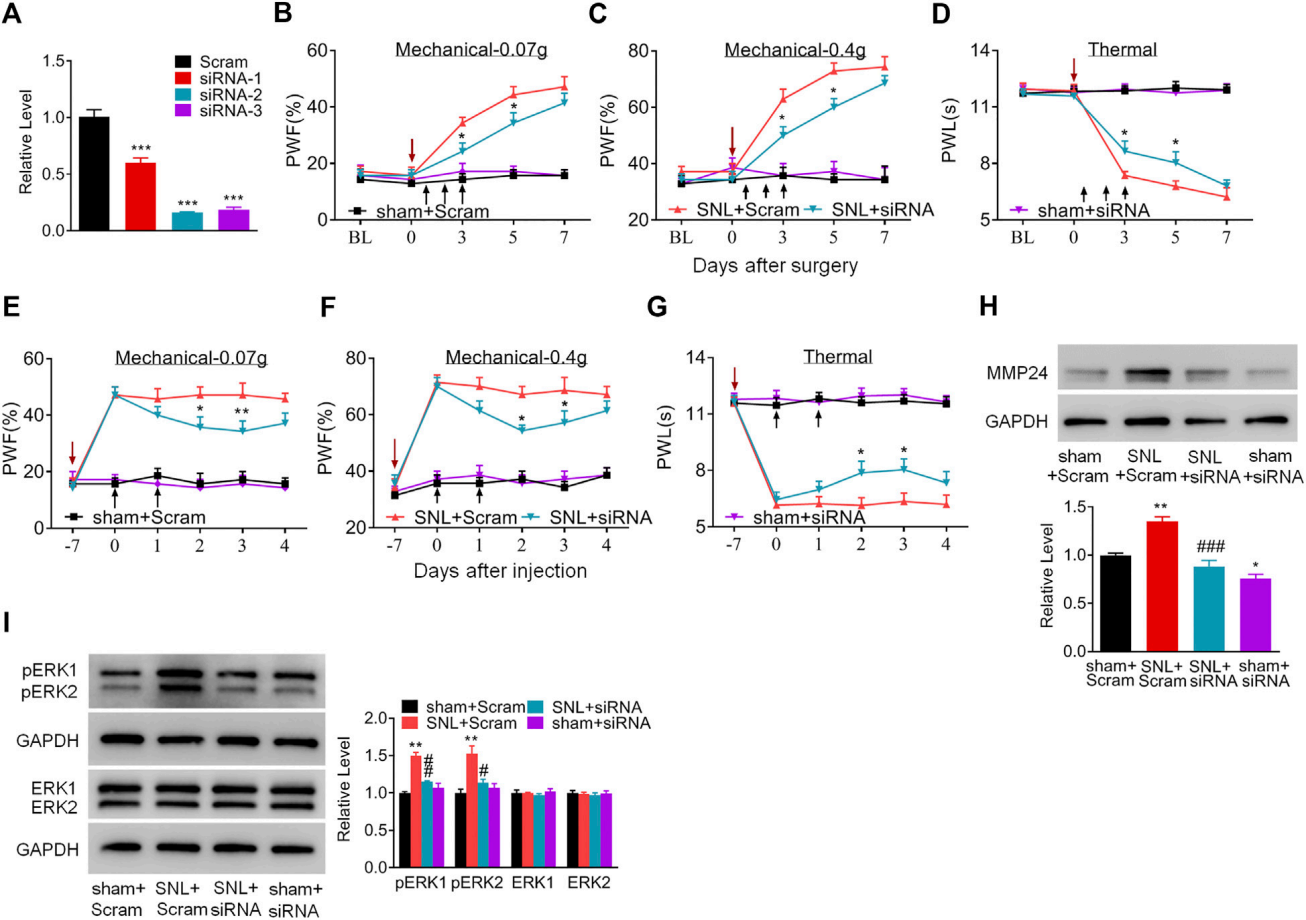
Blocking the increased spinal MMP24 attenuates SNL-induced pain hyperalgesia **(A)**. Effect of three *Mmp24* siRNAs on the expression of *Mmp24* mRNA, among which siRNA-2 shows the best knockdown effect in the cultured spinal cord cells. *n* = 3 repeats, One-way ANOVA followed by *post hoc* Tukey test. F_group_ (3, 8) = 92.87, ^***^ *p* < 0.001 vs. scrambled siRNA (Scram) group. **(B–D)**. Effect of intrathecal injection of *Mmp24* siRNA (siRNA) or control scrambled siRNA (Scram) into the sham or SNL mice on the paw withdrawal responses to mechanical **(B, C)** and heat stimuli **(D)** from day 3 to 7 after SNL as indicated. *n* = 7 mice/group. Two-way RM ANOVA followed by *post hoc* Tukey test. F_group_ (3, 90) = 48.41 for **(B)**, F_group_ (3, 90) = 58.89 for **(C)**, F_group_ (3, 90) = 120.0 for **(D)**. ^*^ *p* < 0.05 vs. the SNL plus Scram group. The red arrow indicates surgery day and black arrow indicates siRNA intrathecal injection. **(E–G)**. Effect of intrathecal injection of *Mmp24* siRNA (siRNA) or control scrambled siRNA (Scram) into the sham or SNL mice on the paw withdrawal responses to mechanical **(E, F)** and heat stimuli **(G)** 7 days after SNL as indicated. *n* = 7 mice/group. Two-way RM ANOVA followed by *post hoc* Tukey test. F_group_ (3, 90) = 53.66 for **(E)**, F_group_ (3, 90) = 61.53 for **(F)**, F_group_ (3, 90) = 136.7 for **(G)**. ^*^ *p* < 0.05, ^**^ *p* < 0.01 vs. the SNL plus Scram group. The red arrow indicates surgery day and black arrow indicates siRNA intrathecal injection. **(H)**. MMP24 protein expression in the spinal cord of sham or SNL mice intrathecally injected with *Mmp24* siRNA (siRNA) or scrambled siRNA (Scram) on day 10 after SNL (2 days after the last siRNA injection). *n* = 3 mice/group. One-way ANOVA followed by *post hoc* Tukey test. F_group_ (3, 8) = 29.00, ^*^ *p* < 0.05, ^**^ *p* < 0.01 vs. sham plus Scram group. ^###^ *p* < 0.001 vs. SNL plus Scram group. **(I)**. Effect of *Mmp24* siRNA or scrambled siRNA on SNL-induced increase in the phosphorylation of ERK1/2 in the spinal cord on day 10 after SNL or sham surgery (2 days after the last siRNA injection). *n* = 3 mice/group. One-way ANOVA followed by *post hoc* Tukey test. F_group_ (3, 8) = 29.00 for pERK1, F_group_ (3, 8) = 11.72 for pERK2. ^**^ *p* < 0.01 vs. the sham plus Scram group; ^#^ *p* < 0.05, ^##^ *p* < 0.01 vs. the SNL plus Scram group.

Does the increased MMP24 in the spinal cord participate in nerve injury-induced pain hypersensitivity? To this end, we first examined the effect of blocking spinal MMP24 increase on the development of SNL-induced pain hypersensitivity. As shown in Figure 2A, siRNA-2 displayed the best knockdown effect compared to the scrambled siRNA (Scram) in the cultured spinal cord cells. Thus, siRNA-2 (*Mmp24* siRNA) and its control scrambled siRNA were intrathecally injected into the sham or SNL mice. Intrathecal injection of *Mmp24* siRNA attenuated SNL-induced mechanical allodynia as demonstrated by a decrease in PWF to mechanical stimuli and ameliorated SNL-induced thermal hyperalgesia as indicated by the increase in PWL to heat stimulation from day 3 to 5 compared to the scrambled siRNA-treated SNL mice (Figures 2B–D). No changes were observed in basal mechanical and heat responses in sham mice following injection with either siRNA (Figures 2B–D). Moreover, we also observed the role of MMP24 in the maintenance phase of neuropathic pain through intrathecal injection of *Mmp24* siRNA or scrambled siRNA into the sham or SNL mice 7 days after SNL. Consistently, blunted mechanical allodynia and heat hyperalgesia were observed on days 9 and 10 after SNL from the *Mmp24* siRNA-treated mice compared to the scrambled siRNA-treated mice (Figures 2E–G). The basal pain behavior of the sham mice (Figures 2E–G) and the locomotor functions of the siRNA-treated mice were not affected (Table 2). As expected, a noticeable decrease in the amount of MMP24 protein was detected in the spinal cord from the *Mmp24* siRNA-treated mice compared with the scrambled siRNA-treated mice on day 10 after SNL (Figure 2H). Consistently, intrathecal injection of *Mmp24* siRNA dampened the SNL-induced spinal neuronal sensitization as indicated by abolishing the SNL-induced increase in pERK1/2 in the spinal cord (Figure 2I). Together, the results described above demonstrate that spinal MMP24 is necessary for SNL-induced central sensitization and pain hypersensitivities.

**TABLE 2:** Locomotor functions.

Treatments	Functional test		
	Placing	Grasping	Righting
LV-*Gfp*	5 (0)	5 (0)	5 (0)
LV-*Mmp24*	5 (0)	5 (0)	5 (0)
*Mmp24* siRNA + SNL	5 (0)	5 (0)	5 (0)
*Mmp24* siRNA + sham	5 (0)	5 (0)	5 (0)
Scrambled siRNA + sham	5 (0)	5 (0)	5 (0)
Scrambled siRNA + SNL	5 (0)	5 (0)	5 (0)

Scores for placing, grasping and righting reflexes were based on counts of each normal reflex exhibited in five trials. All values are Mean (SEM). *n* = 6 mice/group.

### MMP24 Overexpression Causes Neuropathic Pain-like Symptoms

**FIGURE 3:**
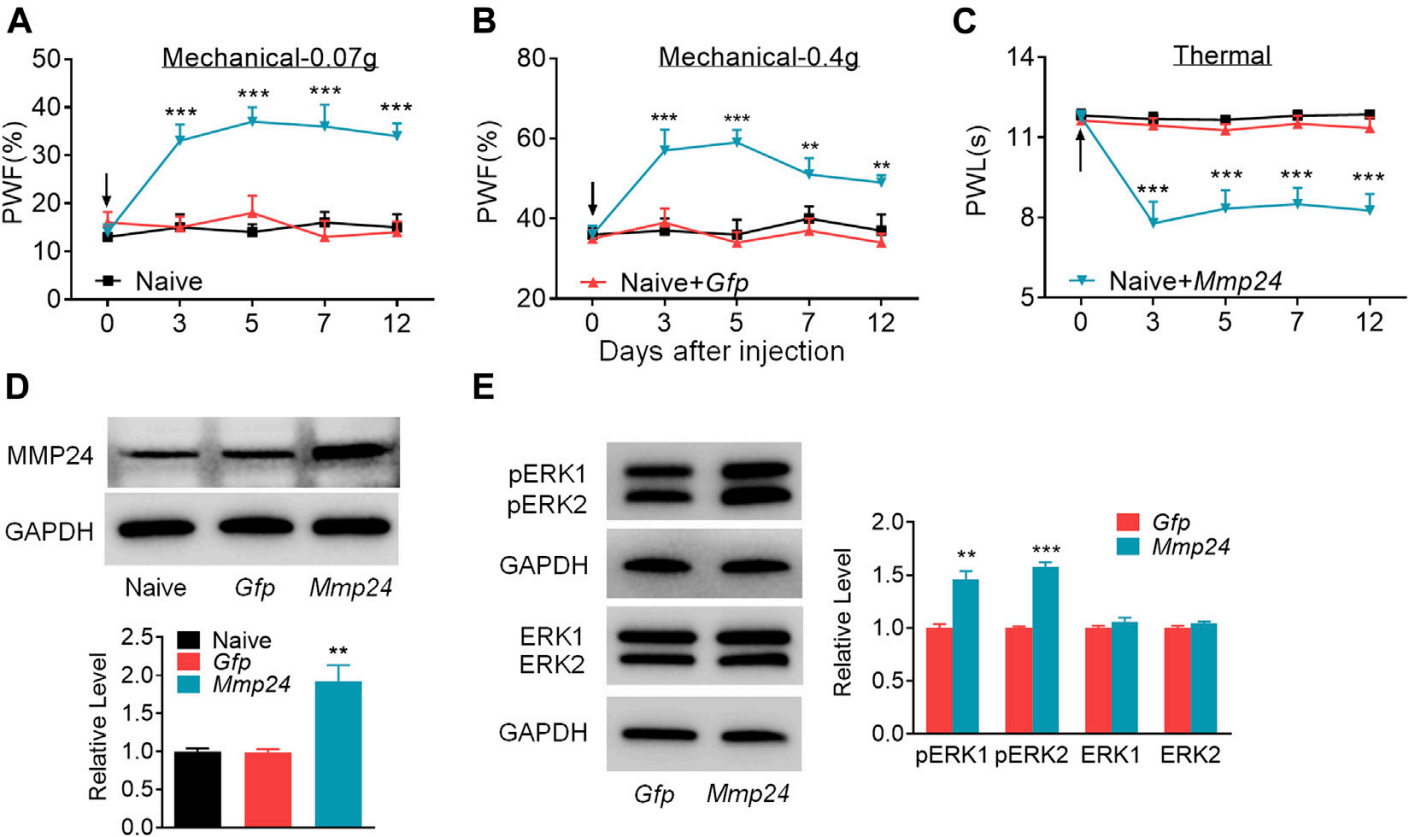
Spinal MMP24 overexpression triggers pain hyperalgesia **(A–C)**. Effect of intraspinal injection of LV-*Mmp24* (*Mmp24*) or LV-*Gfp* (*Gfp*) into the naïve mice on the paw withdrawal responses to mechanical **(A, B)** and heat stimuli **(C)** on day 3, 5, 7, and 12 after injection. *n* = 10 mice/group. Two-way RM ANOVA followed by *post hoc* Tukey test. F_group_ (2, 90) = 56.05 for **(A)**, F_group_ (2, 90) = 29.72 for **(B)**, F_group_ (2, 90) = 69.17 for **(C)**. ^**^ *p* < 0.01, ^***^ *p* < 0.001 vs. the Naive plus *Gfp* group. Black arrow indicates intraspinal virus injection. **(D)**. MMP24 protein expression in the spinal cord of naïve mice 12 days after intraspinal injection with LV-*Mmp24* (*Mmp24*) or LV-*Gfp* (*Gfp*). *n* = 3 mice/group. One-way ANOVA followed by *post hoc* Tukey test. F_group_ (2, 6) = 16.82, ^**^ *p* < 0.01 vs. *Gfp* group. **(E)**. pERK1/2 and ERK1/2 expression in the spinal cord of naïve mice 12 days after intraspinal injection with LV-*Mmp24* (*Mmp24*) or LV-*Gfp* (*Gfp*). *n* = 3 mice/group. ^**^ *p* < 0.01, ^***^ *p* < 0.001 vs. the *Gfp* group by two-tailed unpaired Student’s *t*-test.

We then asked whether the increased MMP24 in the spinal cord would be sufficient to induce neuropathic pain? To this end, we performed intraspinal lentivirus (LV) injection that expressed full-length *Mmp24* (LV-*Mmp24*) into naïve mice. As shown in Figures 3A–C, LV-*Mmp24*, but not its control LV-*Gfp*, induced mechanical allodynia as indicated by an increase in PWF to mechanical stimulation and heat hyperalgesia as demonstrated by a decrease in PWL to heat stimulation from day 3 to 12 post-injection. The locomotor functions were not affected after viral injection (Table 2). Expectedly, the level of MMP24 protein in the L4 spinal cord was significantly increased 12 days after intraspinal injection with LV-*Mmp24* compared to that of LV-*Gfp* (Figure 3D). These behavioral observations were further supported by the following evidence of spinal dorsal horn central sensitization. The level of pERK1/2 was markedly elevated in the L4 spinal dorsal horn on day 12 after intraspinal injection with LV-*Mmp24* compared to that of LV-*Gfp* (Figure 3E).

### m^6^A Modification in the Spinal *Mmp24* mRNA Was Decreased under Neuropathic Pain Condition

**FIGURE 4:**
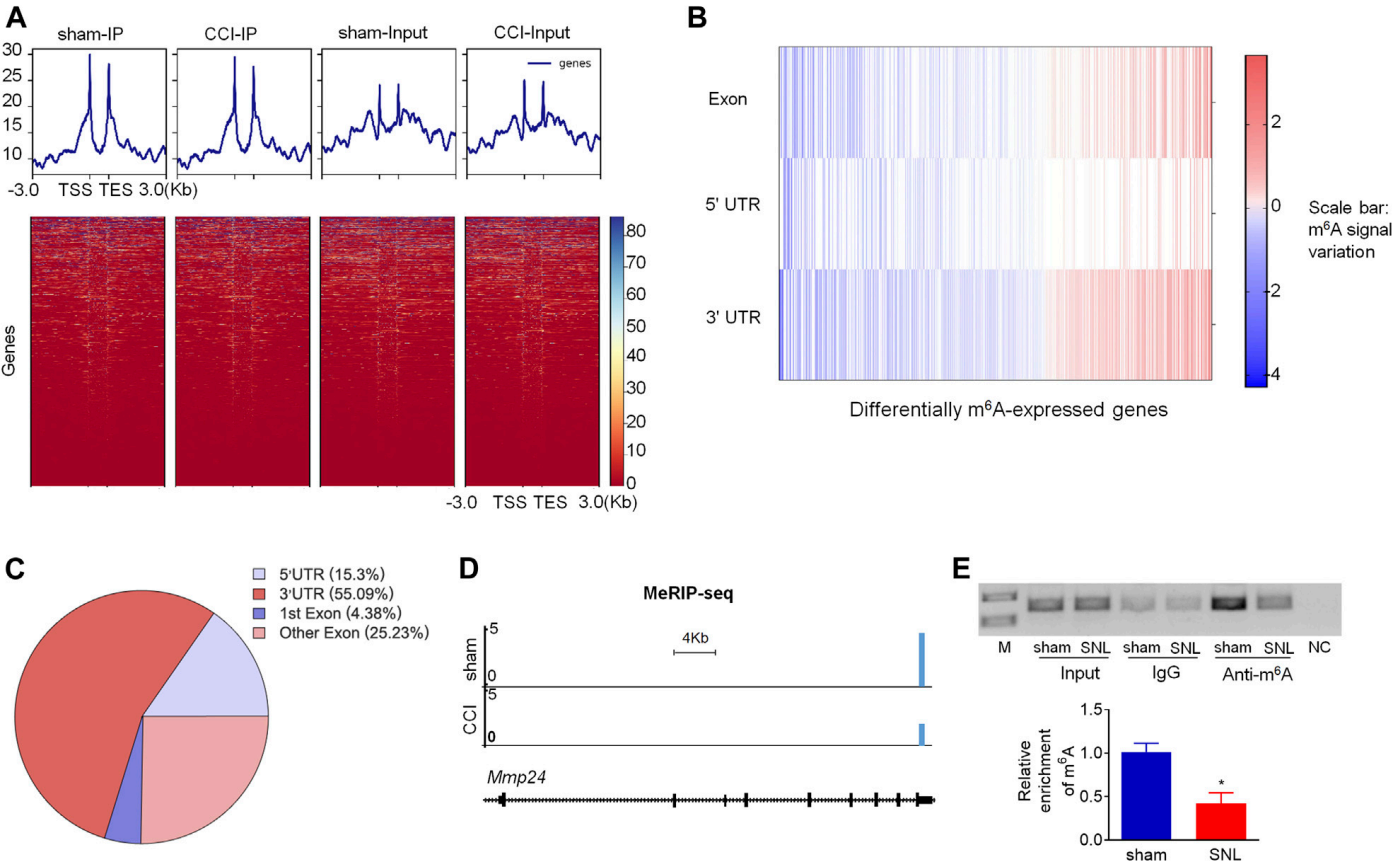
m^6^A modification in spinal *Mmp24* mRNA is decreased after SNL **(A)**. Heatmap of the MeRIP-seq signals from the spinal cord after chronic constriction injury (CCI) or sham surgery. Mean-normalized MeRIP-seq densities of equal bins along the gene. 3-kb region flanking the TSS or the TES are plotted. Red to blue color gradient of the heatmap represents the relative m^6^A level. **(B–C)**. Dynamic changes **(B)** and distribution **(C)** of m^6^A sites across transcripts from the spinal cord on day 7 after CCI or sham surgery. **(D)**. MeRIP-seq assay showed the example tracks of *Mmp24* mRNA from the spinal cord on day 7 after CCI or sham surgery. m^6^A sites (blue) are indicated at the 3′-UTR. **(E)**. Level of *Mmp24* mRNA fragments immunoprecipitated by mouse anti-m^6^A in the spinal cord on day 7 after SNL or sham surgery. Input: total purified fragment. *n* = 4 biological repeats (4 mice/repeat). M: ladder marker. NC: negative control (H2O). ^*^ *p* < 0.05 vs. the sham group by two-tailed unpaired Student's *t*-test.

Although *Mmp24* mRNA remained unchanged after SNL, the MMP24 protein was significantly increased in the spinal cord (Figures 1A,B), suggesting that the translation efficiency of spinal *Mmp24* mRNA may be elevated under neuropathic pain condition. Of note, RNA m^6^A modification is known to play an important role in mRNA translation (He and He, 2021). To this end, we carried out methylated RNA immunoprecipitation sequencing (MeRIP-seq) assay to observe the changes of m^6^A sites across the transcriptome in the spinal cord on day 7 after peripheral nerve injury (Figure 4A). Consistent with previous studies in the DRG (Li et al., 2020), m^6^A sites change after nerve injury occurred predominantly at the 3′-UTR (55.09%) and to lesser extents at coding regions (29.61%), and 5′-UTR (15.3%) (Figures 4B,C). Approximately 55.6% (1,910/3,437) transcripts exhibited a loss of m^6^A sites, 33.9% (1,165/3,437) transcripts exhibited a gain of m^6^A sites, and 10.5% (362/3,437) transcripts exhibited both a loss and gain of m^6^A sites compared to the sham group (Figures 4B,C). Notably, *Mmp24* mRNA exhibited a considerable loss of m^6^A sites at the 3′-UTR (Figure 4D). We then performed RIP-PCR to validate the MeRIP-seq results. The immunoprecipitation identified the m^6^A enrichment of the *Mmp24* mRNA fragments with anti-m^6^A in the spinal cord (Figure 4E). However, the activity of immunoprecipitation from the spinal cord on day 7 post-SNL was significantly decreased compared to the sham group (Figure 4E). In summary, we found that m^6^A modification in the *Mmp24* mRNA was decreased in the spinal cord under neuropathic pain conditions, suggesting the role of m^6^A modification in regulating MMP24 translation.

### m^6^A Modification-Related Genes Expression in the Spinal Cord after SNL

**FIGURE 5:**
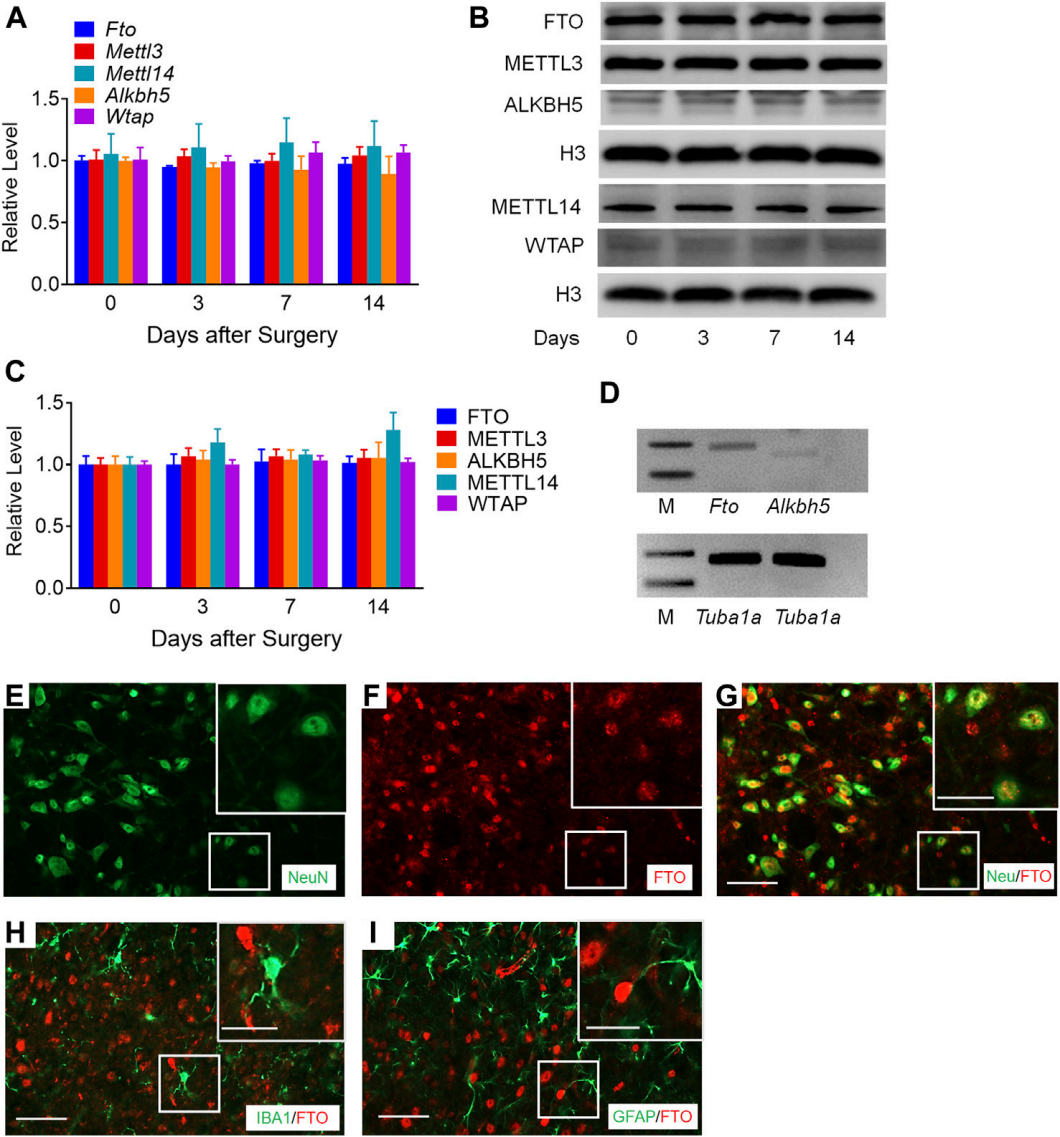
m^6^A-related genes expression in the spinal cord after SNL **(A)**. Expression of *Fto*, *Mettl3*, *Alkbh5*, *Mettl14*, and *Wtap* mRNAs in the spinal cord from day 3 to 14 after SNL. n = 3–5 mice/group/time point. **(B–C)**. Expression of nuclear FTO, METTL3, ALKBH5, METTL14 and WTAP proteins in the spinal cord from day 3 to 14 after SNL. *n* = 7–13 mice/group/time point. **(D)**. The relative expression of *Fto* and *Alkbh5* mRNA in the spinal cord. *n* = 3 repeats. M: ladder marker. **(E–I)**. Representative images of double staining between FTO and markers of neuron, astrocytes and microglia in the spinal dorsal horn. Scale bar: 50 μm; 25 μm (insets).

To determine the specific regulators responsible for the m^6^A modification in the *Mmp24* mRNA, we first examined the expression of m^6^A modification-related methyltransferases, including METTL3, METTL14, and WTAP, and demethylases, including FTO and ALKBH5 in the spinal cord under neuropathic pain condition. Unexpectedly, none of *Mettl3*, *Mettl14*, *Wtap*, *Fto*, and *Alkbh5* mRNAs showed an obvious change in the spinal cord from day 3 to 14 after SNL (Figure 5A). To further check the nucleus and cytoplasm distribution of the protein above, we extracted the nucleus protein of the spinal cord and found that none of the METTL3, METTL14, WTAP, FTO, and ALKBH5 protein exhibited a significant change from day 3 to 14 after SNL (Figures 5B,C). FTO was recently reported to play a vital role in neuropathic pain genesis by diminishing the m^6^A enrichment in pain-related mRNAs in the DRG neurons (Li et al., 2020). Furthermore, FTO expression was found enriched in the spinal cord relative to that of DRG or cortex (Li et al., 2020), and higher than the other demethylase ALKBH5 in the spinal cord (Figure 5D), suggesting the possible role of spinal FTO in neuropathic pain genesis. Likewise, as shown in Figures 5E–I, FTO in the spinal dorsal horn was also expressed in the neurons but not in microglia or astrocytes. Therefore, we hypothesize that FTO may be accountable for the m^6^A modification of *Mmp24* mRNA in the spinal neurons.

### Spinal FTO Is Responsible for the Decreased m^6^A Enrichment and Increased Translation of MMP24 after SNL in the Spinal Cord Neurons

**FIGURE 6:**
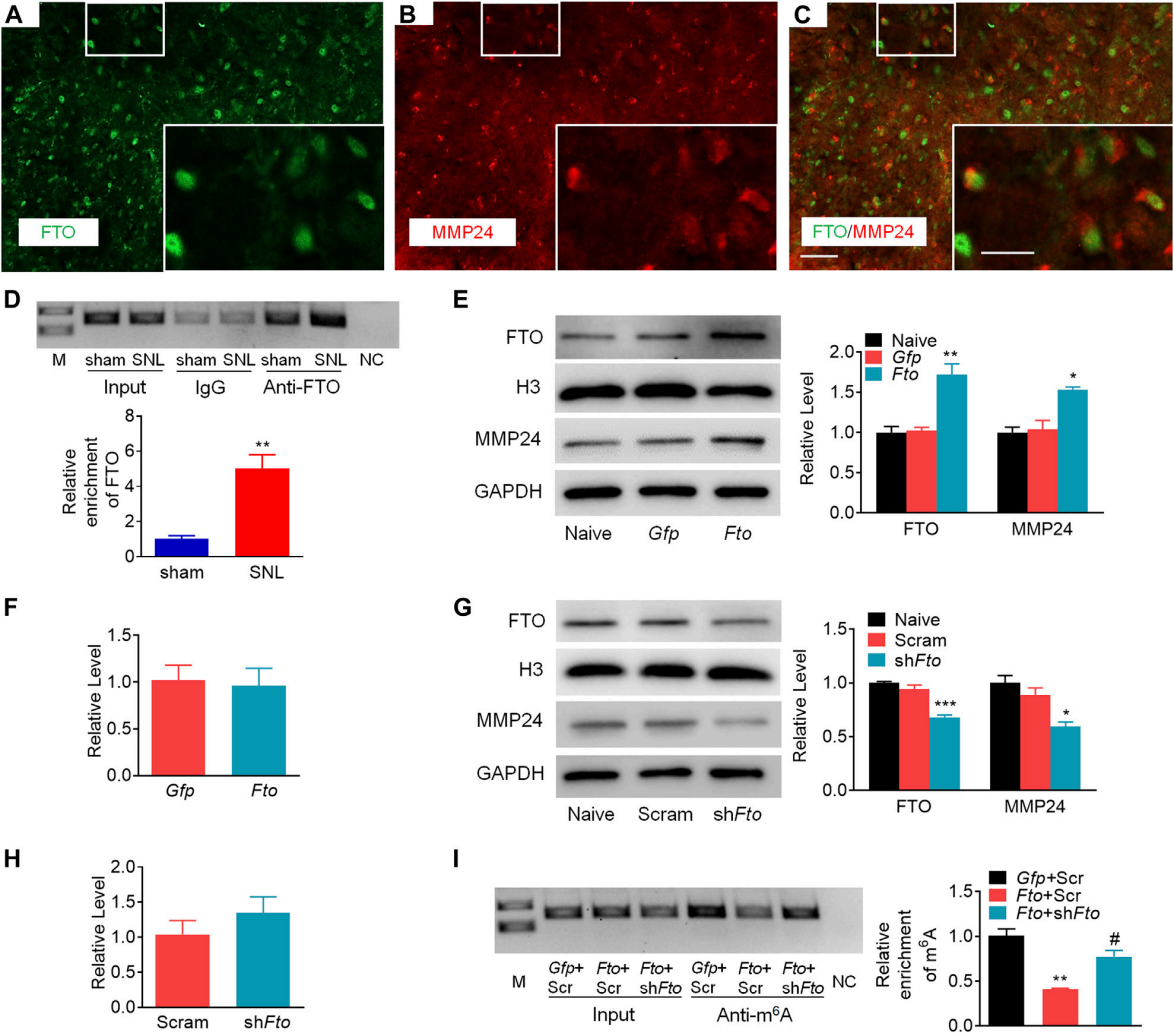
FTO promotes the expression of MMP24 in the spinal neurons **(A–C)**. Representative images of the colocalization between FTO and MMP24 in the spinal dorsal horn. Scale bar: 50 μm; 25 μm (insets). **(D)**. Level of *Mmp24* mRNA fragments immunoprecipitated by mouse anti-FTO in the spinal cord on day 7 after SNL or sham surgery. Input: total purified fragment. *n* = 3 biological repeats (4 mice/repeat). M: ladder marker. NC: negative control (H2O). ^**^ *p* < 0.01 vs. the sham group by two-tailed unpaired Student’s *t*-test. **(E)**. Expression of FTO and MMP24 in the cultured spinal neurons on day 3 after transduction with AAV5-*Gfp* (*Gfp*) or AAV5-*Fto* (*Fto*). *n* = 3 repeats. One-way ANOVA followed by *post hoc* Tukey test. F_group_ (2, 6) = 19.98 for FTO, F_group_ (2, 6) = 14.37 for MMP24. ^*^ *p* < 0.05, ^**^ *p* < 0.01 vs. the *Gfp* group. **(F)**. Expression of *Mmp24* mRNA in the cultured spinal neurons on day 3 after transduction with AAV5-*Gfp* (*Gfp*) or AAV5-*Fto* (*Fto*). *n* = 3 repeats **(G)**. Expression of FTO and MMP24 in the cultured spinal neurons on day 3 after transduction with AAV5-*Fto* shRNA (sh*Fto*) or AAV5-scrambled shRNA (Scram). *n* = 3 repeats. One-way ANOVA followed by *post hoc* Tukey test. F_group_ (2, 6) = 46.71 for FTO, F_group_ (2, 6) = 12.07 for MMP24. ^*^ *p* < 0.05, ^***^ *p* < 0.001 vs. the Scram group. **(H)**. Expression of *Mmp24* mRNA in the cultured spinal neurons on day 3 after transduction with AAV5-*Fto* shRNA (sh*Fto*) or AAV5-scrambled shRNA (Scram). *n* = 3 repeats **(I)**. Enrichment of *Mmp24* mRNA fragments immunoprecipitated by anti-m^6^A from the cultured spinal neurons transduced viruses as shown. *Gfp*: AAV5-*Gfp*; Scr: AAV5-scrambled shRNA; *Fto*: AAV5-*Fto*; sh*Fto*: AAV5-*Fto* shRNA. n = 3 biological repeats/group. One-way ANOVA followed by *post hoc* Tukey test. F_group_ (2, 6) = 24.41. ^**^ *p* < 0.01 vs. the *Gfp* plus Scr group. ^#^ *p* < 0.05 vs. the *Fto* plus Scr group.

To demonstrate the role of FTO in the regulation of MMP24, we first checked the distribution pattern of FTO and MMP24. Double immunostaining showed the colocalization of FTO and MMP24 in the spinal dorsal horn (Figures 6A–C). Moreover, the RIP assay further revealed that FTO could bind to the *Mmp24* mRNA from the spinal cord (Figure 6D), and there was a significant elevation in the binding activity from the spinal cord 7 days after SNL (Figure 6D). It indicates that the SNL-induced decrease in the m^6^A enrichment in *Mmp24* mRNA may be due to the increased FTO occupation in the *Mmp24* mRNA after SNL.

We further examined the effect of FTO on the expression of MMP24 in the cultured spinal neurons. The expression of FTO was first verified 3 days after transduction with AAV5 that expressed full-length *Fto* (AAV5-*Fto*) or *Gfp* (AAV5-*Gfp*) (Figure 6E). A significant increase in the level of MMP24 protein was observed 3 days after transduction with AAV5-*Fto* compared to the AAV5-*Gfp*-treated group (Figure 6E). Interestingly, the level of *Mmp24* mRNA was not altered on day 3 after transduction with AAV5-*Fto* compared to AAV5-*Gfp* (Figure 6F). We further checked the effect of FTO knockdown on the expression of MMP24 in the cultured spinal neurons through the transduction of AAV5 that expressed *Fto* shRNA (AAV5-*Fto* shRNA) or control shRNA (AAV5-scrambled shRNA). The knockdown effect of FTO was confirmed 3 days after transduction of AAV5-*Fto* shRNA (Figure 6G). As expected, the MMP24 protein was markedly downregulated 3 days after transduction with AAV5-*Fto* shRNA compared with that of AAV5-scrambled shRNA (Figure 6G). However, the level of *Mmp24* mRNA was not significantly altered in AAV5-*Fto* shRNA-treated group compared to the AAV5-scrambled shRNA-treated group (Figure 6H), suggesting that FTO may promote the translation of *Mmp24* mRNA in the spinal neurons. We further transduced AAV5-*Fto* into cultured spinal neurons and found that FTO overexpression produced a marked loss of m^6^A enrichment in the *Mmp24* mRNA compared to the control group (Figure 6I). This decrease was reversed by blocking FTO overexpression in the cultured spinal neurons co-transduced with AAV5-*Fto* shRNA (Figure 6I). Given the role of m^6^A modification in mRNA translation (Jia et al., 2019; Zhang M. et al., 2020; Zhang Z. et al., 2020; Liu et al., 2020; Song et al., 2020), the evidence described above indicates that FTO likely erased the m^6^A enrichment in *Mmp24* mRNA to promote the translation of *Mmp24* mRNA in the spinal cord neurons under neuropathic pain condition.

## Discussion

**FIGURE 7:**
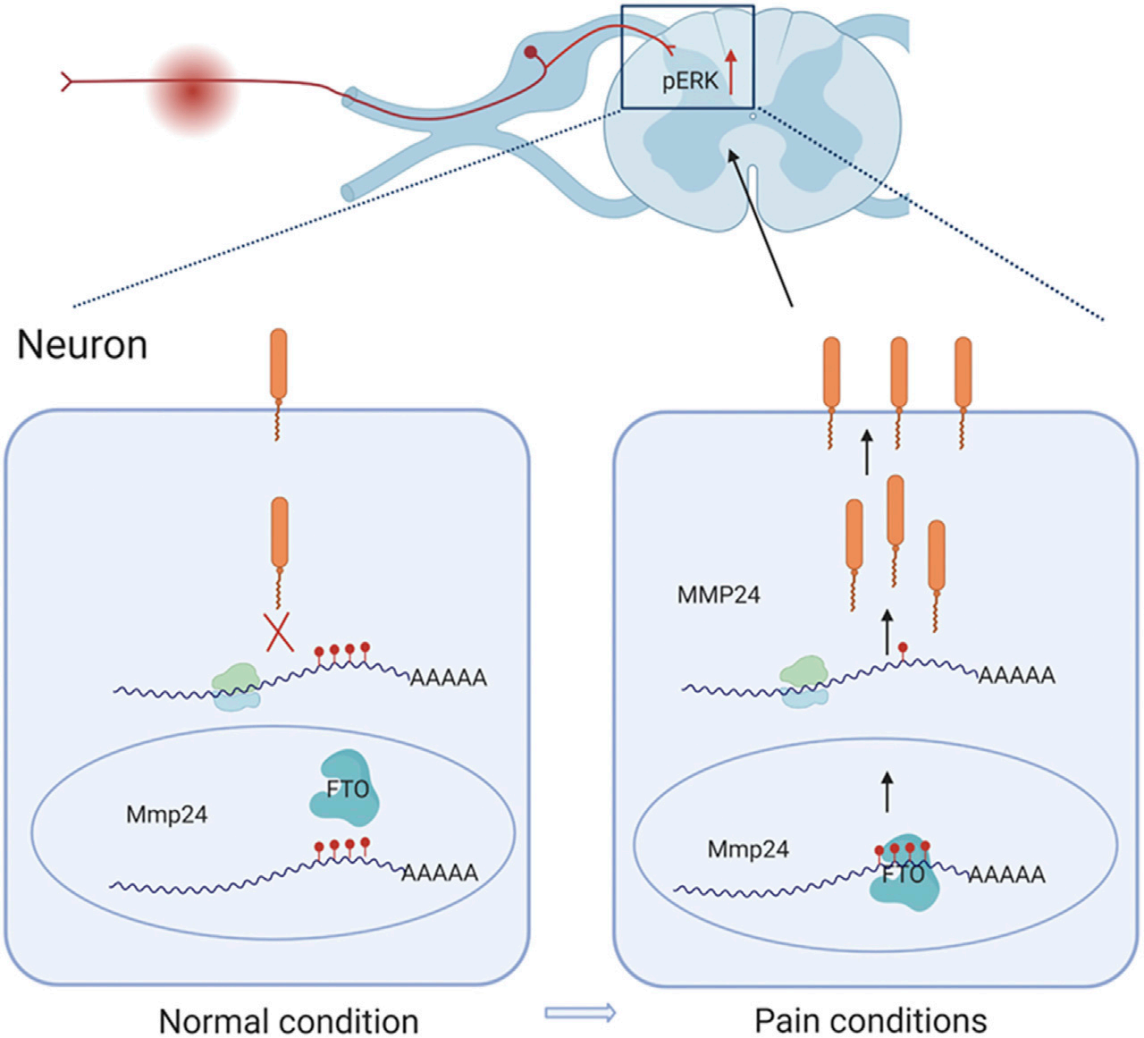
Schematic representation of the mechanism of MMP24 participation in neuropathic pain. Black arrows depict promotion. Red arrows represent upregulation. Never injury can facilitate the binding of FTO to *Mmp24* mRNA in spinal neurocytes nuclei and subsequently lead to the reduced level of m^6^A modification followed by the enhanced translation of its mRNA. Increased MMP24 can facilitate ERK phosphorylation in the spinal cord and induce neuropathic pain.

This study demonstrates that SNL results in an accelerated translation of *Mmp24* mRNA in the spinal cord. Increased spinal MMP24 contributes to the SNL-induced central sensitization and nociceptive hyperalgesia. Mechanistically, SNL leads to an elevation of FTO occupation in the *Mmp24* mRNA. Increased occupation of FTO contributes to reducing m^6^A enrichment in *Mmp24* mRNA in the spinal neurons after SNL, which subsequently accelerates the translation of MMP24 and eventually contributes to neuropathic pain by activating ERK (Figure 7).

MMP24 was explicitly detected in neurons of both the central and peripheral nervous systems (Hayashita-Kinoh et al., 2001; Sekine-Aizawa et al., 2001) and identified as a brain-specific MMP (Llano et al., 1999; Wang and Pei, 2001). The present study showed that MMP24 was mainly expressed in the spinal neurons as it was co-expressed with NeuN-labeled individual neurons. Meanwhile, our results also supported the existence of MMP24 in the spinal astrocytes and microglia after SNL even though at a low abundance. Besides, the morphologic observation of the MMP24-labeled cells proved the presence of MMP24 in the matrix of the dorsal horn, supporting the role of MMP24, as an extracellular matrix metalloproteinase, in regulating cleavage of the cell-cell adhesion molecule N-cadherin to influence the neuronal circuit formation and plasticity (Komori et al., 2004; Folgueras et al., 2009).

Previous studies showed that mice with genetically ablated MMP24 exhibited a lowered severity of mechanical allodynia after partial sciatic nerve injury or spinal cord transection (Komori et al., 2004) through the lowered degradation of chondroitin sulfate proteoglycans, which are abundant in neuronal tissues and inhibit neurite outgrowth (Dou and Levine, 1994). Deletion of *Mmp24* also relieved thermal pain after the inflammation model through increased interaction between mast cells and nociceptive neurites (Folgueras et al., 2009). Our study further demonstrated the involvement of spinal MMP24 in the different phases of neuropathic pain. Specifically, blocking the SNL-induced increase in MMP24 in the spinal cord attenuates both the mechanical allodynia and heat hyperalgesia in both development and maintenance phases, accompanied by lessened activation ERK1/2. Moreover, intraspinal specific overexpression of MMP24 by LV persistently induced both the mechanical allodynia and heat hyperalgesia with the activation of ERK1/2 absence of SNL. MMP24 was reported to show the ability to proteolytically activate MMP2 (Llano et al., 1999), which could cleave pro-IL-1β to activate ERK in the spinal cord (Kawasaki et al., 2008). Furthermore, MMP24 was required for the inflammatory response to TNF-α and IL-1β in the peripheral nervous system (Folgueras et al., 2009). Thus, MMP24 is likely to activate ERK through inflammatory mediators like IL-1β and TNF-α in the spinal cord. ERK1/2 phosphorylation has been widely considered in recent years as a spinal neuron sensitization marker (Zhuang et al., 2005; Gu et al., 2018). Its activation could subsequently induce the expression and release of various downstream pain-related genes like *Cxcl10*, *Ccl2*, and *Ccl7*, or affect neuronal excitability of the central neurons, and eventually trigger the pain behaviors (Jiang et al., 2020a; Jiang et al., 2020b).

Mounting evidence indicates that epigenetic alterations play a critical role in neuropathic pain induction and maintenance, including histone acetylation/methylation, DNA methylation and non-coding RNAs (Penas and Navarro, 2018). Spinal coactivator-associated arginine methyltransferase 1 (CARM1) regulated histone methylation of the potassium channel promoter to participate in neuropathic pain development (Hsieh et al., 2021). Also, spinal ten-eleven translocation methylcytosine dioxygenase 1 (Tet1) decreased the DNA methylation state in metabotropic glutamate receptor subtype 5 (mGluR5) promoter to mediate spinal plasticity and pain hypersensitivity (Hsieh et al., 2017). Epigenetic modification at the transcriptional level has achieved much progress in neuropathic pain genesis these years, while the translational level epigenetic regulation is still incomplete. Interestingly, the current study demonstrates that although the level of *Mmp24* mRNA was not altered in SNL, the expression of MMP24 protein was significantly increased in the spinal cord in a time-dependent manner, suggesting the accelerated translation of spinal *Mmp24* mRNA after SNL. A previous study showed that m^6^A enrichment in many transcripts was changed in the DRG upon peripheral nerve injury. This change contributed to increased translation during axon regeneration, revealing the critical role of m^6^A modification in response to nerve injury (Weng et al., 2018). Our MeRIP-seq and RIP-PCR assay showed a considerable loss of m^6^A sites in *Mmp24* mRNA in the spinal cord under neuropathic pain. It suggests that m^6^A modification in *Mmp24* mRNA may be accountable for the accelerated translation of *Mmp24* mRNA. Indeed, we found that after SNL, the increased level of MMP24 protein (not *Mmp24* mRNA) was accompanied by a higher occupancy of FTO in the *Mmp24* mRNA and the consequently lessened enrichment of m^6^A. Moreover, FTO overexpression erased the m^6^A sites in the *Mmp24* mRNA and increased the level of MMP24 protein. Conversely, FTO knockdown markedly increased the m^6^A enrichment in the *Mmp24* mRNA and reduced the expression of MMP24 protein, but not *Mmp24* mRNA, in the spinal neurons. Together, the accelerated *Mmp24* mRNA translation after SNL in the spinal cord was likely resulted from the lowered FTO-mediated m^6^A modification in *Mmp24* mRNA.

Previous studies displayed that FTO was localized in the nucleus in neurons in the central nervous system, including the hippocampus and midbrain (Hess et al., 2013; Li et al., 2017). Functionally, FTO bound to the m^6^A sites and reduced the m^6^A enrichment to mediate the biogenesis of its target transcripts in the central neurons (Hess et al., 2013; Leonetti et al., 2020). Consistently, the present study revealed that FTO was co-expressed with NeuN in nuclei of the spinal neurons and colocalized with MMP24. However, the expression of spinal FTO was not altered after SNL. How the FTO preferentially binds to *Mmp24* mRNA under the neuropathic pain condition is unclear. This could be due to that the binding of FTO and *Mmp24* mRNA in the spinal cord is regulated by nerve injury-sensitive signaling pathways (Bresson et al., 2020), such as the upregulation of mediators in promoting the interaction of FTO and *Mmp24* mRNA or downregulation of competitors in competing for the binding between FTO and *Mmp24* mRNA (Song et al., 2019; Ontiveros et al., 2020). Although demethylase FTO, as an “eraser”, removed the m^6^A in *Mmp24* mRNA after SNL, the specific “reader” decoding the m^6^A modification in the *Mmp24* mRNA remains unknown. Previous studies found that m^6^A promoted translation efficiency via the reader proteins (Roundtree et al., 2017), like YTHDF1 (Jia et al., 2019). However, recent studies from C.H. et al. found that the effect of m^6^A on translation was highly heterogeneous and depended on the binding of specific RNA binding proteins (Zhang Z. et al., 2020). For example, the newly identified m^6^A effector YBX3, opposite to YTHDF1 in function, mediated m^6^A effect by repressing the translation of YBX3-bound m^6^A transcripts (Snyder et al., 2015; Zhang Z. et al., 2020). Here, the m^6^A loss-mediated promotion in *Mmp24* mRNA translation possibly depends on the effectors like YBX3, but not YTHDF1. Further investigation is warrented for its identification.

In summary, our study reveals an FTO-triggered epigenetic mechanism of MMP24 upregulation in the spinal cord after SNL. Blocking the SNL-induced increase of MMP24 in the spinal cord mitigated pain hypersensitivity both in the development and maintenance phase without altering the basal/acute responses or locomotor functions. MMP24 may be an endogenous initiator of neuropathic pain and could be a potential target for this disorder's prevention and treatment.

## Data Availability

The raw data supporting the conclusions of this article will be made available by the authors, without undue reservation.
